# Stability of Outer Membrane Vesicles-Based Vaccines, Identifying the Most Appropriate Methods to Detect Changes in Vaccine Potency

**DOI:** 10.3390/vaccines9030229

**Published:** 2021-03-06

**Authors:** Elena Palmieri, Vanessa Arato, Davide Oldrini, Beatrice Ricchetti, Maria Grazia Aruta, Werner Pansegrau, Sara Marchi, Fabiola Giusti, Ilaria Ferlenghi, Omar Rossi, Renzo Alfini, Carlo Giannelli, Gianmarco Gasperini, Francesca Necchi, Francesca Micoli

**Affiliations:** 1 GSK Vaccines Institute for Global Health (GVGH) S.r.l., Via Fiorentina 1, 53100 Siena, Italy; elena.x.palmieri@gsk.com (E.P.); vanessa.x.arato@gsk.com (V.A.); davide.x.oldrini@gsk.com (D.O.); b.ricchetti88@gmail.com (B.R.); maria-grazia.x.aruta@gsk.com (M.G.A.); omar.x.rossi@gsk.com (O.R.); renzo.x.alfini@gsk.com (R.A.); carlo.x.giannelli@gsk.com (C.G.); gianmarco.x.gasperini@gsk.com (G.G.); francesca.x.necchi@gsk.com (F.N.); 2 GSK, Via Fiorentina 1, 53100 Siena, Italy; werner.x.pansegrau@gsk.com (W.P.); sara.x.marchi@gsk.com (S.M.); fabiola.x.giusti@gsk.com (F.G.); ilaria.x.ferlenghi@gsk.com (I.F.)

**Keywords:** outer membrane vesicles, GMMA, stability, vaccine

## Abstract

Ensuring the stability of vaccines is crucial to successfully performing global immunization programs. Outer Membrane Vesicles (OMV) are receiving great attention as vaccine platforms. OMV are complex molecules and few data have been collected so far on their stability. OMV produced by bacteria, genetically modified to increase their spontaneous release, simplifying their production, are also known as Generalized Modules for Membrane Antigens (GMMA). We have performed accelerated stability studies on GMMA from different pathogens and verified the ability of physico-chemical and immunological methods to detect possible changes. High-temperature conditions (100 °C for 40 min) did not affect GMMA stability and immunogenicity in mice, in contrast to the effect of milder temperatures for a longer period of time (37 °C or 50 °C for 4 weeks). We identified critical quality attributes to monitor during stability assessment that could impact vaccine efficacy. In particular, specific recognition of antigens by monoclonal antibodies through competitive ELISA assays may replace in vivo tests for the potency assessment of GMMA-based vaccines.

## 1. Introduction

Outer Membrane Vesicles (OMV) are spherical bi-layered membrane structures with a diameter in the range of 20–250 nm, naturally released by Gram-negative bacteria during their growth [1]. They are primarily made of bacterial outer membrane constituents, therefore containing key antigenic components to elicit an immune response [2]. OMV also contain lipopolysaccharides (LPS), and other pathogen-associated molecular patterns [3], have optimal size for cellular uptake and display antigens in their native conformation and orientation, representing a promising vaccine platform [4,5]. Indeed, a meningitis serogroup B vaccine, which contains a bacterial OMV component, was approved in 2013 for human use [6] and many other vaccine candidates based on this technology are under development, targeting different diseases [7].

Bacteria can be genetically manipulated in order to enhance OMV release [8], resulting in hyperblebbing microorganisms whose blebs have been also called GMMA (Generalized Modules for Membrane Antigens). Additional genetic modifications are generally introduced to reduce LPS reactogenicity, most often by modifying the lipid A structure of LPS molecules [9,10]. GMMA production process is simple and highly cost-effective [8,11] which makes GMMA attractive for the development of vaccines against neglected diseases. This technology has been applied in fact to different pathogens having a huge burden of disease in low- and middle-income countries, like invasive non-typhoidal *Salmonella* (iNTS) [12,13,14], *Neisseria meningitidis* [15], and *Shigella* serotypes [8,16], with the most advanced candidate tested up to phase 1 and 2 clinical trials [17,18,19]. In clinical trials, to further enhance their tolerability, GMMA have been formulated with aluminum hydroxide, which lowers GMMA’s pyrogenic response in rabbits [8].

An important aspect to take into account during the development of a vaccine is its thermal stability. Indeed, especially in impoverished settings, where it is difficult to maintain the cold chain, developing a thermostable product would be a great advantage. For this reason, it is important that thermostability is given priority early in the development of new vaccines, making efforts in the identification of stabilizing formulations when needed. OMV composition is complex and the interaction of all their different components (e.g., phospholipids, LPS and outer membrane proteins) with each other through various non-covalent forces determine their physico-chemical properties and overall stability. Diverse degradation processes may occur simultaneously during storage of OMV vaccines: not only the proteins and other key components, such as LPS, can undergo chemical or conformational changes, but also the OMV structure may change or even be destroyed [20].

Accelerated stability studies are useful to evaluate the stability profile of a candidate vaccine, and the availability of analytical methods able to detect changes in vaccine physico-chemical properties impacting efficacy of the vaccine is crucial. With this in mind, we started to investigate GMMA stability under very harsh conditions, in order to test the ability of methods in place to reveal eventual changes occurring in GMMA quality attributes. We then evaluated the impact of milder thermal stability conditions for longer periods of time and performed immunogenicity studies in mice to understand the effect of observed changes on the resulting immune response elicited by GMMA. The aim was to identify critical quality attributes to monitor over time, with the final future goal of possibly replacing in vivo studies with a method in vitro for vaccines potency testing.

## 2. Materials and Methods

### 2.1. GMMA Production

GMMA were produced at 30 L scale from mutant strains of *Salmonella enterica* serovar Typhimurium isolate 2192 (Δ*tolR*::*aph*, Δ*msbB*::*tet*, Δ*pagP*::*cat*, LT-2 collection, University of Calgary) [21], *Salmonella enterica* serovar Enteriditis isolate SA618 (Δ*tolR*::*aph*, Δ*msbB*::*tet*, Δ*pagP*::*cat*, CEESA EASSA II collection of Quotient Bioresearch Limited) [21], *Shigella flexneri* 2a 2457T (-pINV, Δ*tolR*::*aph*, Δ*msbB*::*cat*, Public Health England) and *Shigella sonnei* 53G (Δ*tolR*::*aph*, Δ*msbB*::*erm*, Δ*msbB2*::*cat* Δ*virG*::*nadAB*, Walter Reed Army Institute of Research) [8,22] and purified as previously reported [8]. Purified GMMA were stored in saline at—70 ± 10 °C.

### 2.2. GMMA Formulation on Alhydrogel

Bulk GMMA formulations were prepared by adsorbing *S.* Typhimurium and *S.* Enteritidis GMMA diluted to 80 μg/mL (O-antigen, OAg, based concentration) and *S. sonnei* and *S. flexneri* 2a GMMA diluted to 26.7 μg/mL (OAg based concentration) in NaCl 154 mM NaH_2_PO_4_ 20 mM pH 6.5 on Alhydrogel at the final concentration of 0.7 mg/mL Al^3+^.

### 2.3. Stability Studies

GMMA samples, both unformulated (drug substance) and formulated on Alhydrogel (drug product), were stressed as follows: harsh temperature stress conditions (e.g., 100 °C for 40 min) were applied to *S*. Typhimurium, *S.* Enteriditis and *S. sonnei* GMMA; mild temperature stress conditions for longer periods of time (e.g., 37 °C and 50 °C for 4 weeks) were applied to *S.* Typhimurium, *S*. Enteriditis, *S. flexneri* 2a and *S. sonnei* GMMA. Samples were incubated in an oven in the stability conditions mentioned above. GMMA drug substance were stressed at the concentration of approximately 3 mg/mL (protein based concentration), in saline or in phosphate buffer pH 6.5 (after buffer exchange through centrifugal ultrafiltration using Amicon Ultra filter with a membrane cut-off of 100 kDa). GMMA drug product were stressed at the bulk concentration.

OAg was extracted from *S.* Typhimurium and *S. flexneri* 2a GMMA as previously described [12] and resulting samples were heated at 37 °C and 50 °C for 4 weeks in water at the concentration of 9 and 3 mg/mL, respectively.

### 2.4. GMMA Characterization

#### 2.4.1. GMMA Drug Substance Characterization

GMMA drug substance were characterized through a panel of methods previously described [12,23,24,25,26]. In particular, GMMA particles size was determined by Dynamic Light Scattering (DLS) and High Performance Liquid chromatography-Size Exclusion Chromatography/Multiangle Light Scattering (HPLC-SEC MALS) [24]. Purity was assessed by HPLC-SEC analysis [12]; total protein content was estimated by micro BCA using bovine serum albumin (BSA) as a reference following the manufacturer’s instructions (Thermo Scientific, Waltham, MA, USA). Protein pattern profile was analyzed by Sodium Dodecyl Sulphate-Polyacrylamide Gel Electrophoresis (SDS-PAGE) analysis [12]. OAg sugar content and composition were determined by High-Performance Anion-Exchange Chromatography coupled to Pulsed Amperometric Detector (HPAEC-PAD) [12,23,26].

OAg identity and quantification was also performed by competitive enzyme-linked immunosorbent assay (cELISA) [25], using specific monoclonal antibodies (mAb), produced by Takis from hybridoma derived from mice immunized with the respective GMMA. Coating antigens used in cELISA were the following: *S.* Typhimurium OAg (at the concentration of 5 µg/mL in carbonate buffer pH 9.6), *S*. Enteritidis OAg (at the concentration of 15 µg/mL in carbonate buffer pH 9.6), *S. sonnei* LPS (at the concentration of 0.5 µg/mL in PBS pH 7.2) and *S. flexneri* 2a OAg (at the concentration of 0.5 µg/mL in carbonate buffer pH 9.6). IC50 was defined as the OAg concentration of the competitor (GMMA or purified OAg) needed to inhibit the binding of a specific mAb to the coating antigen on the ELISA plate by 50%. IC50 fold variation in stressed OAg or GMMA samples was calculated with respect to the IC50 value of the reference controlsample.

OAg was extracted from GMMA samples through acid hydrolysis and analyzed by HPLC-SEC for length determination; the amount of core reducing end KDO (2-keto-3-deoxy-octonate) was assumed equal to the amount of lipid A and quantified by semicarbazide/HPLC-SEC method after sugar extraction [26]. OAg O-acetylation level was estimated via ^1^H NMR [26]. Supernatants post GMMA ultracentrifugation (4 °C, 30 min, 110,000 rpm, rotor K factor 15) were analyzed by HPLC-SEC analysis for the evaluation of released OAg in stressed GMMA samples and by semicarbazide/HPLC-SEC method to verify if free OAg derived from the cleavage of KDO-lipid A linkage.

Lipid A structures were analyzed using Matrix-Assisted Laser Desorption/Ionization-Mass Spectrometry (MALDI-MS), after performing a mild hydrolysis of LPS and precipitation of lipid A to preserve its structure (i.e., pyrophosphate groups). GMMA at 1 mg/mL in protein were kept at 100 °C for 1 h in presence of 40 mM acetate buffer pH 4.5 and 3% N-Octyl-β-D-gluco-pyranoside in screw-cap vials. After centrifugation, lipid A pellet was washed with 500 µL of water and resuspended in a 4:1 mixture of chloroform:methanol and assayed as previously reported [9].

GMMA samples were analyzed through Differential Scanning Calorimetry (DSC) analysis at protein concentration of 1 mg/mL and thermograms were acquired with Microcal VP-Capillary DSC (Malvern Panalytical) using the following experimental conditions: scan rate 150 °C/h, temperature range 10–120 °C, filter period 5 s.

GMMA were also observed in Transmission Electron Microscopy (TEM) with negative staining method, with the following procedure: 5 μL of diluted GMMA sample (80 ng/µL) were loaded onto glow-discharged copper grids (3 grids/each sample). The excess of sample was blotted away using filter paper and immediately negatively-stained by adding 5 μL of the stain solution (NanoW, Nanoprobes Inc., Yaphank, NY, USA) for 30 s. The excess was blotted away and the grids left dry at room temperature for few minutes. Samples were observed using a FEI TECNAI G2 Spirit transmission microscope operating at 100 kV, equipped with an 2 K × 2 K CCD Veleta.

#### 2.4.2. GMMA Drug Product Characterization

Alhydrogel-formulated GMMA were checked for particles size through a laser diffraction technique using Mastersizer 3000 (Malvern Panalytical, Malvern, Worcestershire, UK), setting the refractive index (1.53), the absorption (0.01) and the density (1), the type of material (non-spherical particle type) and the refractive index of the dispersant (1.33). A total of Six measurements for each sample were recorded, with a duration of 30 s for the background and of 30 s for the sample (both for blue and red light) with obscuration between 13 and 17. During measurements, the sample dispersion was maintained in the cell (Hidro SV, Malvern Panalytical, Malvern, Worcestershire, UK) by stirring at 1000 rpm. Data were processed through General Purpose model algorithm.

GMMA adsorption on Alhydrogel was verified through SDS-PAGE analysis with silver staining detection following manufacturer’s instruction (SilverQuest Silver Staining kit, ThermoFisher Scientific) performed on formulation supernatants, separated from Alhydrogel by two sequential centrifugations (18,000 rcf, 15 min, 4 °C).

OAg identity and quantification were established by Formulated Alhydrogel cELISA (FAcE), as previously described [25]. Same mAb, coating antigens described for cELISA were used and same data analysis was performed.

### 2.5. Immunogenicity Studies in Mice

*S.* Typhimurium and *S. flexneri* 2a GMMA (drug substance or drug product) were put in stability at their bulk concentration and diluted with saline or Alhydrogel diluent at the concentrations tested in the immunogenicity studies in mice conducted at the end of the stability periods.

Animal study with *S.* Typhimurium GMMA stressed in harsh conditions was performed at Toscana Life Science Animal Care Facility under the animal project 479/2017-PR 09/06/2017, approved by the Italian Ministry of Health; the other animal experiments were conducted at Charles River Laboratories in accordance with good animal practice as defined by the relevant international (Directive of the European Parliament and of the Council on the Protection of Animals Used for Scientific Purposes, Brussels 543/5) and local animal welfare guidelines.

A number of female CD1 mice from Charles River, 4–6 weeks old, were immunized intraperitoneally with 200 μL of vaccine at days 0 and 28. Sera were collected at days 27 and 42. Eight mice per group were injected with GMMA with (*S.* Typhimurium) or without (*S.* Typhimurium and *S. flexneri* 2a) Alhydrogel (Aluminum hydroxide at 0.7 mg/mL Al^3+^). Complete adsorption of GMMA on Alhydrogel was confirmed by SDS-PAGE and silver staining analysis of supernatants from the different formulations. GMMA were tested at different OAg doses.

Individual mouse sera were tested for anti-OAg and anti-GMMA proteins total IgG by ELISA, as previously described [21], using as coating antigens *S.* Typhimurium OAg (at the concentration of 5 µg/mL in carbonate buffer), OAg-negative GMMA from *S. enterica* serovar Typhimurium isolate 1418 Δ*tolR*::FRT Δ*rfbU-P*::*aph* (at the concentration of 5 µg/mL in PBS) and *S. flexneri* 2a OAg (at the concentration of 0.5 µg/mL in carbonate buffer).

Single sera were also tested against wild-type bacterial strains in serum bactericidal assay (SBA) based on luminescent readout [27,28], as previously described [16]. Results of the assay were expressed as the IC50, the reciprocal serum dilution that resulted in a 50% reduction of luminescence and thus corresponding to 50% growth inhibition of the bacteria present in the assay. GraphPad Prism 7 software was used for curve fitting and IC50 determination. Titers below the minimum measurable signal were assigned a titer of 50, corresponding to half of the first dilution of sera tested.

### 2.6. Statistical Analysis

Statistical analysis was performed using GraphPad Prism 7. Dose–response relationships were evaluated through Spearman’s rank correlation. The parallelism of dose–response curves was assessed by the parallel line method: when the slopes of the curves for stressed and control formulations obtained by log-transforming ELISA or SBA results vs. log transformed antigen doses were not significantly different from each other, comparison of the Y-intercepts was performed.

## 3. Results

### 3.1. GMMA Stressed at 100 °C

We started to investigate GMMA stability under very harsh conditions, to verify if the analytical methods in place for GMMA characterization (Table 1) were able to detect any possible occuring change in GMMA and Alhydrogel-formulated GMMA.

GMMA derived from three different pathogens, *S.* Typhimurium, *S.* Enteriditis and *S. sonnei*, were incubated at 100 °C for 40 min in their storage buffer (e.g., saline) and the stressed materials were characterized in comparison to the controls. All GMMA tested have the OAg portion of LPS molecules as key ingredient [29,30], so in-depth characterization of such component was performed.

**Figure 1:**
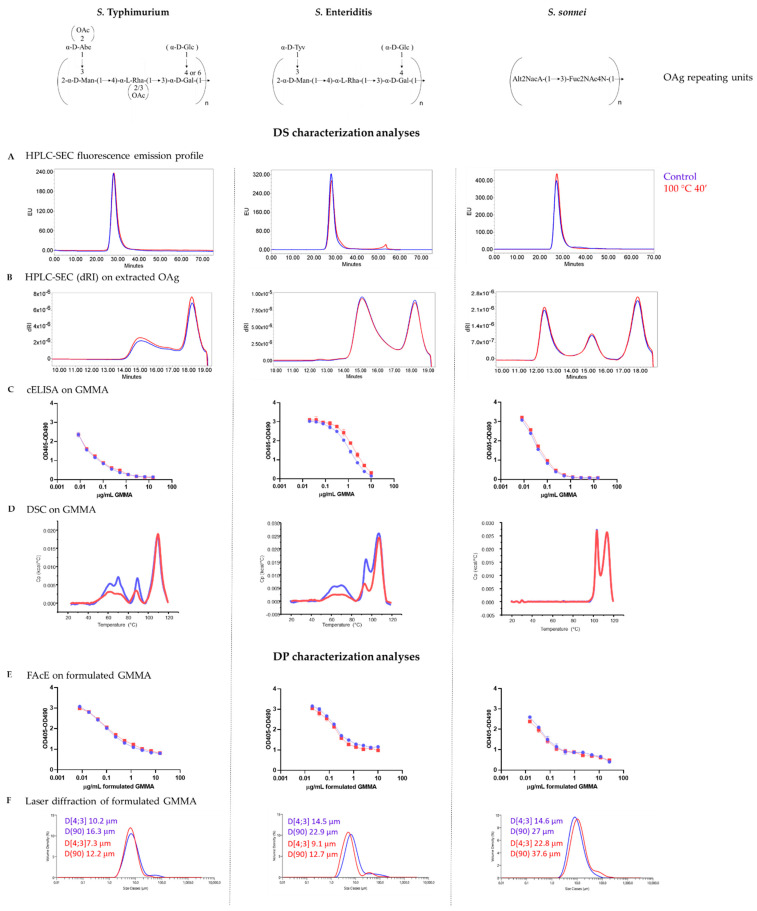
Stability of *S*. Typhimurium, *S*. Enteriditis and *S. sonnei* GMMA, both drug substance (**A**–**D**) and drug product (**E**,**F**), under very harsh conditions (100 °C for 40 min). No change in HPLC-SEC fluorescence emission profiles of stressed GMMA (**A**) and in OAg size populations distribution (**B**). No differences in anti-OAg specific mAb recognition for all three different types of GMMA, both drug substance (**C**) and drug product (**E**). Evidence of protein denaturation occurring in iNTS GMMA (**D**). No changes detected in terms of particle size (**F**) when GMMA were stressed after formulation on Alhydrogel. Blu lines refer to control GMMA, red lines to stressed GMMA.

For all three different GMMA examined, we did not observe any major change in terms of fluorescence emission profile by HPLC-SEC analysis (Figure 1A), particle size, measured both by DLS and HPLC-SEC MALS, OAg to protein ratio (Table S1) and OAg length (Figure 1B). Additionally, OAg O-acetylation level in *S.* Typhimurium and *S.* Enteriditis GMMA (iNTS GMMA) was not impacted by these stress conditions (Table S1). We also verified that lipid A maintained its penta-acylated structure in iNTS GMMA stressed samples (Figure S1). By cELISA we saw that there were no differences between stressed and control GMMA in their ability to compete with the coated OAg for the binding of anti-OAg specific mAb (Figure 1C).

DSC analysis revealed instead that protein denaturation occurred irreversibly after 40 min of incubation at 100 °C in iNTS GMMA, as shown by the reduction of heat capacity (Cp) in the transition peak between 80 °C and 100 °C, but this was not observed in *S. sonnei* GMMA (Figure 1D).

The same stress conditions were applied to iNTS and *S. sonnei* GMMA formulated with Alhydrogel, and also in this case we did not observe any difference in anti-OAg specific mAb recognition between stressed and control formulated GMMA through FAcE (Figure 1E). We also verified that GMMA remained adsorbed on Alhydrogel after thermal stress (no signal detected in drug product supernatants by SDS-PAGE silver staining), maintaining substantially the same size (Figure 1F).

**Figure 2:**
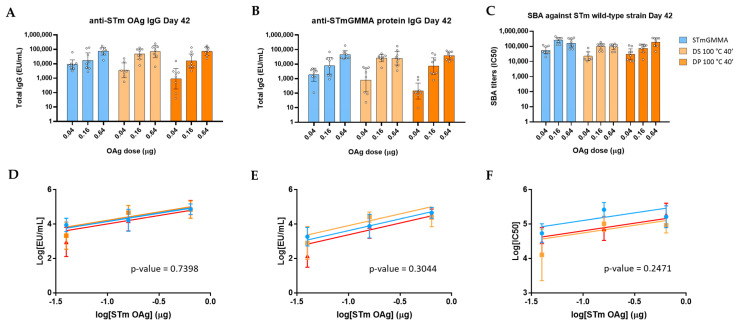
Immunogenicity results from a dose-ranging study in mice with S. Typhimurium GMMA stressed in harsh temperature conditions: (**A**) anti-OAg IgG by ELISA, (**B**) anti-GMMA protein IgG by ELISA and (**C**) SBA against *S.* Typhimurium wild-type strain induced by control GMMA formulated on Alhydrogel and GMMA stressed at 100 °C for 40 min before (in the legend, DS, drug substance, 100 °C 40′) or after (DP, drug product, 100 °C 40′) formulation on Alhydrogel. Single mice EU/mL in ELISA and SBA titers (dots) are reported together with geomean for each group (bars) and 95% confidence interval. Parallel line analysis showed that Y-intercepts of the dose–response curves of the three different formulations were not significantly different among each other in the case of anti-OAg (**D**), anti-GMMA protein ELISA (**E**) and SBA titers (**F**), being their *p*-values above the specified alpha of 0.05.

We then investigated the effect of harsh temperature stress on the immune response induced by *S.* Typhimurium GMMA formulated with Alhydrogel, stressed before (drug substance 100 °C 40′) or after being formulated (drug product 100 °C 40′), in a dose-ranging immunogenicity study in mice. We verified a dose–response relationship for all GMMA formulations (anti-OAg IgG, anti-GMMA proteins IgG and SBA titers) in the range of doses selected, 2 weeks after two immunizations at 4-week interval time. No significant differences were observed among all GMMA formulations in terms of anti-OAg IgG response (Figure 2A) and SBA titers (Figure 2C). Interestingly, anti-GMMA proteins IgG response, measured by using OAg-negative GMMA as coating antigen, was also not affected (Figure 2B) independently from proteins denaturation occurring, as verified by DSC analysis (Figure 1D). Ability of GMMA to boost anti-OAg and anti-GMMA proteins IgG responses (comparing response induced 4 weeks after the first immunization with response 2 weeks after the second immunization) was also verified through Wilcoxon matched-pairs statistical test (Figure S2).

### 3.2. GMMA Stressed at 37 °C or 50 °C in Saline

GMMA were then stressed at lower temperatures but for longer periods of time. In particular, *S.* Typhimurium and *S. flexneri* 2a GMMA drug substances were incubated in saline at 37 °C and 50 °C for 4 weeks and characterized in parallel to the corresponding control GMMA.

**Figure 3:**
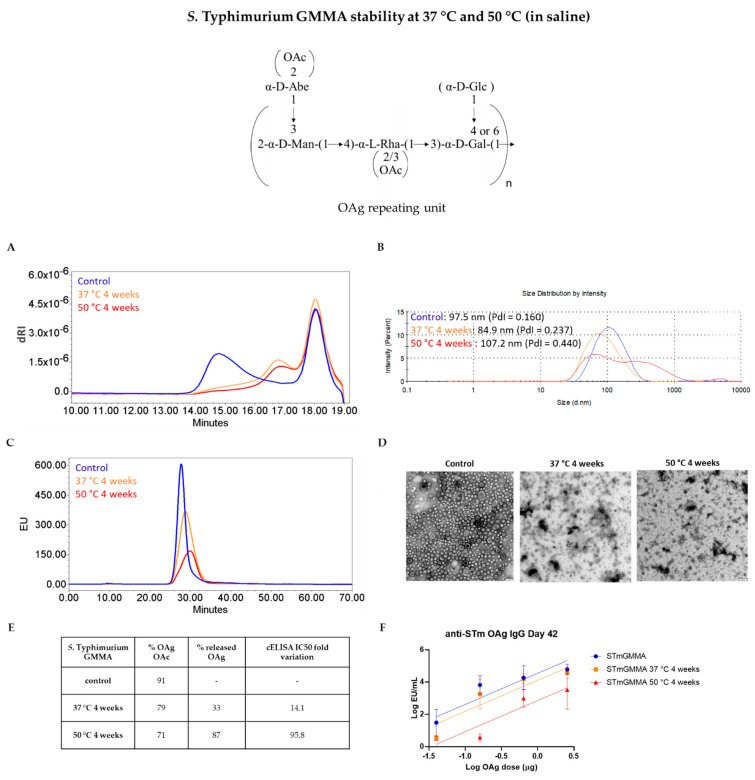
Characterization of *S.* Typhimurium GMMA stressed for 4 weeks at 37 °C or 50 °C: (**A**) HPLC-SEC analysis of extracted OAg from GMMA samples; (**B**) DLS, (**C**) HPLC-SEC and (**D**) TEM analyses performed on GMMA: disruption and aggregation of GMMA start after one month at 37 °C with a worsening trend visible at the higher temperature of 50 °C (scale bar = 100 nm). (**E**) Summary table with OAg O-acetylation levels, percentage of OAg detached from GMMA membrane, and cELISA IC50 fold variation. (**F**) Stressed GMMA were tested in mice: animals were immunized at day 0 and 28 and anti-OAg IgG response determined by ELISA at day 42 in single sera from each group. The graph reports parallel lines (log transformed ELISA units vs. log transformed μg OAg dose) where Y-intercepts of the dose–response curves are compared (*p*-value = 0.0177).

For *S.* Typhimurium GMMA, the storage at 37 °C and 50 °C for 4 weeks caused a change in the OAg length and a slight decrease in the OAg O-acetylation level (Figure 3A,E). Particles aggregation was observed by DLS after 4 weeks at 50 °C, with Z-average diameter increasing from 97.5 to 107.2 nm and accompanied by an increase in the Polydispersity Index (PdI) from 0.160 to 0.440 (Figure 3B). Additionally, the fluorescence emission profile of GMMA in HPLC-SEC analysis was impacted (Figure 3C). TEM analysis confirmed the presence of aggregates. Indeed, GMMA particles started to disrupt and aggregate after a 4-week incubation at 37 °C with severe morphological modifications after incubation at 50 °C (Figure 3D). At the end of the stability period, GMMA were also ultracentrifuged and resulting supernatants were analyzed by HPLC-SEC verifying the presence of OAg chains released from GMMA membranes. More in detail, we estimated an 87% of released OAg in the supernatant of *S.* Typhimurium GMMA incubated at 50 °C and a 33% in that of GMMA stressed at 37 °C (Figure 3E). We verified by HPLC-SEC analysis with semicarbazide method that the cleavage of the OAg chains from LPS molecules anchored on GMMA surface occurred at the level of the linkage between the KDO sugar at the end of the core region and Lipid A (Figure S3). Recognition of stressed GMMA by a specific anti-OAg mAb in cELISA was also affected, as highlighted by the IC50 fold variation values (Figure 3E).

**Figure 4:**
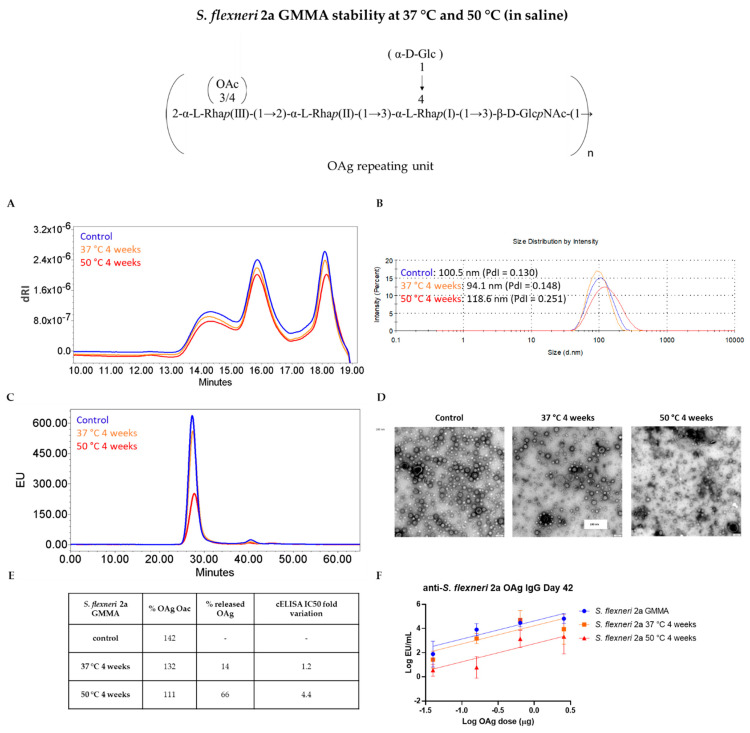
Characterization of *S. flexneri 2a* GMMA stressed for 4 weeks at 37 °C and 50 °C: (**A**) HPLC-SEC analysis of extracted OAg from GMMA samples; (**B**) DLS, (**C**) HPLC-SEC and (**D**) TEM analyses performed on GMMA: disruption and aggregation of GMMA vesicles start after one month at 37 °C with a worsening trend visible at the higher temperature of 50 °C (scale bar = 100 nm). (**E**) Summary table with OAg O-acetylation level, percentage of OAg detached from GMMA membrane and cELISA IC50 fold variation. (**F**) Stressed GMMA were tested in mice: animals were immunized at day 0 and 28 and anti-OAg IgG response determined by ELISA at day 42 in single sera from each group. the graph reports parallel lines (log transformed ELISA units vs. log transformed μg OAg dose) where Y-intercepts of the dose–response curves are compared (*p*-value = 0.0074).

For *S. flexneri* 2a GMMA, similar changes were observed to those for *S.* Typhimurium GMMA stored in same stability conditions: a slight decrease in the OAg O-acetylation level and the presence of released OAg in the supernatants following GMMA ultracentrifugation (Figure 4E), despite in lower amount respect to *S.* Typhimurium GMMA (66% after incubation at 50 °C and 14% after incubation at 37 °C), probably because in this case there was no contribution from OAg length cleavage as occurred in *S.* Typhimurium GMMA. In fact, no changes were observed in OAg chain length (Figure 4A). Again, we observed particles aggregation by DLS and TEM analyses and a decrease in the fluorescence emission intensity of GMMA peak in stressed samples by HPLC-SEC (Figure 4B,D,C, respectively). Recognition of stressed GMMA by a specific anti-OAg mAb in cELISA was once again impacted, in particular at 50 °C (Figure 4E).

We then performed dose-ranging studies to see if changes revealed by physico-chemical and immunological methods could affect the immune response elicited by GMMA in mice. For both *S.* Typhimurium and *S. flexneri* 2a GMMA, a dose–response relationship was found among the groups of mice immunized with increasing OAg doses either of control or stressed GMMA, in terms of anti-OAg IgG, anti-GMMA protein IgG (tested both at day 27 ad 42, Figure S4) and in terms of SBA titers (tested at day 42, Figures S4 and S5). Anti-OAg IgG response 27 days after the first immunization was significantly lower in the case of *S.* Typhimurium GMMA stressed at 37 °C and 50 °C with respect to the control GMMA, while for *S. flexneri* 2a GMMA there was no difference in the response elicited by stressed or control samples. Fourteen days after second injection, only GMMA stressed at 50 °C gave anti-OAg IgG response significantly lower compared to the reference for both types of GMMA (Figure 3F and Figure 4F), paralleling differences observed by cELISA and reflecting changes in GMMA aggregation and percentage of OAg detached from GMMA membranes. SBA titers at day 42 reflected the anti-OAg IgG ELISA results. Differently, anti-*S.* Typhimurium GMMA proteins IgG response was not impacted by the stress conditions tested both at day 27 and day 42 (Figure S4).

### 3.3. GMMA Stressed at 37 °C or 50 °C in Buffer at pH 6.5

Since Alhydrogel-formulated GMMA are at pH 6.5, we decided to evaluate the stability of GMMA derived from different pathogens (*S.* Typhimurium, *S.* Enteriditis, *S. sonnei* and *S. flexneri* 2a) at this pH. GMMA were exchanged in phosphate buffer at pH 6.5 prior incubation at 37 °C and 50 °C for 4 weeks. GMMA formulated with Alhydrogel at the same pH were object of the same stability investigations.

Differently from what observed in saline, no aggregation was seen for all different types of GMMA investigated. We observed instead a slight decrease in particle size distribution (Table 2). We also verified that the decrease in particles size is not a reversible process: indeed, warming up GMMA from 25 °C to 70 °C and then cooling down again to 25 °C, particles size remained smaller than the starting one (Figure S6). Only a slight decrease in fluorescence emission profiles was observed by HPLC-SEC (tested for *S. flexneri* 2a, Figure S7). In *S.* Enteriditis, *S. sonnei* and *S. flexneri* 2a GMMA, the size of the extracted OAg remained unchanged throughout the performed stability study; while *S*. Typhimurium OAg gave a variation of its length distribution with formation of species at lower molecular weight as previously observed in saline (Figure 3A). Lower percentages of OAg resulted detached from GMMA membrane as verified by HPLC-SEC analysis of the supernatants collected after ultracentrifugation (Figure S8). The level of the OAg O-acetylation instead dramatically decreased after 4 weeks at 50 °C both in *S.* Typhimurium and *S. flexneri* 2a GMMA, going from a percentage of 87 and 183 to 15 and 34, respectively (Table 2). *S.* Enteritidis was characterized by low O-acetylation levels from the start. In cELISA, only for *S.* Typhimurium and *S. flexneri* 2a GMMA incubated at 50 °C, we found that specific anti-OAg mAb did no longer bind to the OAg displayed on GMMA after thermal stress (Table 2), reflecting changes observed for corresponding OAg in terms of O-acetylation level.

In parallel, we investigated the stability of the corresponding purified *S.* Typhimurium and *S. flexneri* 2a OAg, to understand if the changes found in some OAg features (e.g., OAg length and/or O-acetylation) were related to the stability of the OAg itself or in the context of GMMA membrane: we observed the same trend in O-acetylation decrease for both *S.* Typhimurium and *S. flexneri* 2a OAg (from 86 and 165 to 21 and 39, respectively, after 4 weeks at 50 °C), followed by a loss of anti-OAg mAb recognition in cELISA (IC50 fold variation of 55 and 81, respectively in the same samples incubated at 50 °C for 4 weeks), while we did not observe any change in *S.* Typhimurium OAg length distribution (Figure S9). Additional studies are ongoing to better understand why *S.* Typhimurium OAg chain length modification happens in the context of GMMA membrane.

Regarding the stability of the same GMMA formulated with Alhydrogel and subjected to the same stress conditions, no impact on GMMA adsorption was found and no major differences were observed in terms of the overall formulation size, except for *S. sonnei* GMMA after a 4-week incubation at 50 °C, whose size grew noticeably with presence of visible particles in suspension (D [4,3] = 26.1 µm and D(90) = 72.6 µm). As for the corresponding unformulated GMMA, *S.* Typhimurium and *S. flexneri* 2a GMMA recognition by their specific anti-OAg mAb in FAcE analysis was impacted after stress at 50 °C (IC50 fold variation greater than 10, Figure S10).

## 4. Discussion

OMV/GMMA have complex composition to deal with for the assessment of their stability and potency. Here, we aimed to verify if analytical methods in place for GMMA characterization (Table 1) were able to detect possible changes impacting vaccine efficacy and to identify critical attributes to monitor during stability studies. Stressed GMMA from different pathogens were tested to verify broad applicability of the results obtained. Surprisingly, we found that heating at 100 °C, even if for a relatively short period of time, did not cause major changes in all GMMA tested. Only protein denaturation was observed, as demonstrated by DSC analysis on *S*. Typhimurium and *S*. Enteriditis GMMA. However, in our study with stressed S. Typhimurium GMMA formulations, we verified that this change does not impact the immunogenicity of protein antigens on GMMA surface. Additionally, the immune response elicited by the OAg, for which the proteins can play a role of carrier, was not affected either after primary immunization or booster. It is instead reported that a heat treatment at 100 °C for 30 min of OMV from *Porphyromonas gingivalis*, which contain both LPS and the FimA fimbriae as their major constituents, abolished the ability of OMV to elicit specific immunoglobulins [31]. When OMV are treated in such drastic conditions, results can be different based on the specific nature of the key antigens delivered.

Stress conditions at lower temperatures and for longer periods of time produced instead detectable physico-chemical changes in GMMA we tested, that likely resulted to be of different nature according to storage buffer pH. In fact, at higher pH (e.g., 6.5), major changes were related to the OAg structure per se. In particular, OAg O-acetylation was impacted as verified for *S.* Typhimurium and *S. flexneri 2a* GMMA. No particles aggregation was observed and a general trend in the diminishing of the hydrodynamic diameter in all different GMMA (*S.* Typhimurium, *S.* Enteriditis, *S. flexneri* 2a and *S. sonnei*) was found. We have verified that such change is irreversible. In other types of OMV, the average particle size gradually decreased after three months of storage at 37 °C or 56 °C [20] too.

At lower pH (5.2–5.4 in saline) GMMA particles stability was instead impacted: OAg chains release from GMMA membrane, as consequence of KDO-lipid A linkage lability [32], led to membrane instability, causing particles disruption and rearrangement in particles of bigger size. In agreement with what we observed, it has been recently reported that acidic pH and salt concentration can induce OMV aggregation in native and lipopolysaccharide modified *E. coli* strains [33]. It is also known that phosphoryl residues in the core region of LPS are critical for outer membrane stability, presumably due to their potential role in stabilizing adjacent LPS molecules through electrostatic interactions with divalent cations [34,35,36,37,38,39]. GMMA we are investigating are produced from strains possessing a penta-acylated lipid A, so membrane stability could be already different from that of GMMA with a wild-type hexa-acylated lipid A [40].

Overall major changes observed were associated to OAg features, such as O-acetylation and length, and particle structural integrity. Interestingly, specific anti-OAg mAb available were able to detect such changes and paralleled responses in mice. This opens the possibility to use cELISA based assays as alternative to in vivo potency studies. In particular, FAcE assay could allow us to check GMMA stability directly on Alhydrogel formulation [25], without the need to desorb GMMA from Alhydrogel to perform any other kind of characterization. FAcE assay will be further evaluated and developed, attempting to identify optimal mAb and proper acceptance ranges.

Although characterized by a prevalent protein composition, differently from GMMA examined in this study that have OAg as key ingredient, meningococcal B OMV stressed at 56 °C also resulted in the total destruction of particle structure and protein conformation with impact on the corresponding immunogenicity [20]. Moreover, when meningococcal B OMV were incubated at 37 °C, the antigenicity decreased to almost zero within 6 months of incubation; instead, at 56 °C, it disappeared within 3 months [20]. No morphological changes were instead observed after incubating *Porphyromonas gingivalis* OMV at 37 °C or 55 °C for 1 h [31], meaning that longer incubation times are required to alter OMV physico-chemical properties. Importantly, it has been reported that OMV-based vaccines are stable for years, both as adsorbed to aluminum-containing adjuvants or as unadsorbed vesicles in bulk at 5 °C [41,42] and real-time stability studies conducted at 4 °C showed that OMV from meningococcal serogroup B strain were stable for at least one year after production [43].

## 5. Conclusions

In conclusion, we have demonstrated that we can monitor changes occurring in GMMA with a comprehensive panel of analytical methods. This is also key in determining the feasibility of controlled temperature chain (CTC) strategy for GMMA-based vaccines, being able with our methods to assess the stability of GMMA when exposed at temperatures above 40 °C for a certain number of days, essential requirement of this new approach to vaccine management. Allowing us to overcome limitations related to cold chain distribution, the CTC approach has the potential to contribute to a broader immunization coverage and equity in low-income countries, representing an important added value in the case of a low-cost vaccine technology focused on global health like GMMA.

Secondly, we have selected cELISA and FAcE assays as a way to predict from appropriately designed accelerated stability tests, the stability of GMMA at 2–8 °C for longer periods of time, allowing to avoid real-time stability studies and a complete characterization of the material, with consequent huge benefits in terms of time and cost savings.

## Figures and Tables

**Table 1:** Panel of analytical methods used for the characterization of GMMA drug substances and drug products, thus formulated with Alhydrogel.

	Product Quality Attribute	Method	Reference
**Drug** **substance**	Purity	HPLC-SEC (fluorescence emission profile, A260–A280 nm)	[12]
	Size and aggregation status	SEC-MALS/DLS	[24]
	OAg identity and quantification	cELISA	[25]
	OAg quantification	HPAEC-PAD	[12,23,26]
	Total protein quantification	Micro BCA	-
	OAg length	HPLC-SEC on extracted OAg	[26]
	OAg O-acetylation content	^1^H NMR on extracted OAg	[26]
**Drug** **product**	OAg identity and quantification	FAcE	[25]
	Size distribution	Laser diffraction	-
	OAg and protein not adsorbed to Alhydrogel	SDS-PAGE silver staining	-

**Table 2:** Characterization of *S*. Typhimurium, *S*. Enteriditis, *S. flexneri* 2a and *S. sonnei* GMMA at pH 6.5 stressed at 37 °C and 50 °C for 4 weeks and compared to control GMMA.

Quality Attribute	Z-Average (d, nm)			OAg/Protein *w*/*w* Ratio			OAg O-Acetylation %				IC50 Fold Variation	
Stability Condition	Control	37 °C4 w	50 °C4 w	Control	37 °C4 w	50 °C4 w	Control	37 °C4 w	50 °C4 w	Control	37 °C4 w	50 °C4 w
**STmGMMA**	93.84 (PdI = 0.188)	80.22 (PdI = 0.223)	82.31 (PdI = 0.262)	0.70	0.71	0.74	87	35	15	-	4.9	164.6
**SEnGMMA**	88.36 (PdI = 0.152)	83.79 (PdI = 0.161)	83.65 (PdI = 0.191)	1.76	1.84	2.01	2.48	1.77	1.23	-	1.8	1.9
***S. flexneri* 2a GMMA**	92.12 (PdI = 0.100)	87.76 (PdI = 0.129)	95.71 (PdI = 0.177)	0.96	0.98	1.06	183	131	34	-	9.2	20.2
***S. sonnei***	127.5 (PdI = 0.175)	116.4 (PdI = 0.187)	100.6 (PdI = 0.178)	0.23	0.23	0.25	-	-	-	-	1.3	1.8

## Data Availability

Not applicable.

## Supplementary Materials

The following are available online at https://www.mdpi.com/2076-393X/9/3/229/s1, Table S1: Characterization of *S.* Typhimurium (STm), *S.* Enteriditis (SEn) and *S. sonnei* GMMA subjected to a harsh temperature stress (100 °C for 40 min) compared to control GMMA; Figure S1: MALDI-TOF-MS analysis on lipid A extracted from *S.* Typhimurium (A) and *S.* Enteriditis GMMA (B) after harsh stress at 100 °C for 40 min in comparison to that of control samples; Figure S2: Immunogenicity results from dose-ranging studies in mice with *S.* Typhimurium stressed in harsh temperature conditions; Figure S3: HPLC-SEC analysis with semicarbazide method performed on undiluted supernatants of stressed *S.* Typhimurium (A) and *S. flexneri* 2a GMMA (B); Figure S4: Immunogenicity results from a dose-ranging study in mice with *S.* Typhimurium stressed at 37 °C or 50 °C for 4 weeks in comparison to control GMMA; Figure S5: Immunogenicity results from a dose-ranging study in mice with *S. flexneri 2a* GMMA stressed at 37 °C or 50 °C for 4 weeks in comparison to controlGMMA; Figure S6: DLS experiment performed warming up *S.* Typhimurium and *S.* Enteriditis GMMA to 70 °C and cooling down to the starting temperature of 25 °C; Figure S7: Comparison between HPLC-SEC fluorescence emission profiles of *S. flexneri* 2a GMMA stored in saline or phosphate buffer at pH 6.5, after a 4-week incubation at 50 °C; Figure S8: HPLC-SEC analysis of the supernatants of *S. flexneri* 2a GMMA stored in saline or phosphate buffer at pH 6.5, after a 4-weeks incubation at 50 °C; Figure S9: Stability of purified *S.* Typhimurium and *S. flexneri* 2a OAg incubated at 37 °C and 50 °C for 4 weeks; Figure S10: FAcE analyses on *S.* Typhimurium (A) and *S. flexneri* 2a GMMA (B) formulations stressed at 37 °C and 50 °C for 4 weeks.
