# Supplementary material for: Stability of Outer Membrane Vesicles-Based Vaccines, Identifying the Most Appropriate Methods to Detect Changes in Vaccine Potency
Source: Vaccines (Basel). 2021 Mar 6;9(3):229. doi: 10.3390/vaccines9030229 (PMC7998687; doi:10.3390/vaccines9030229)
Supplement: Supplementary file 1 [file vaccines-09-00229-s001.pdf]

vaccines

MDPI

Table S1. Characterization of *S.* Typhimurium (STm), *S.* Enteriditis (SEn) and *S. sonnei* GMMA subjected to a harsh temperature stress (100 °C for 40 minutes) compared to control GMMA.

Quality Attribute		Control STmGMMA	STmGMMA 100 °C 40′	Control SEnGMMA	SEnGMMA 100 °C 40′	Control *S. sonnei* GMMA	*S. sonnei* GMMA 100 °C 40′
Particle size	Z-ave (diameter)	95.74 nm (PdI = 0.176)	98.67 nm (PdI = 0.205)	90.39 nm (PdI = 0.161)	89.28 nm (PdI = 0.184)	134.3 nm (PdI = 0.196)	135.2 nm (PdI = 0.219)
	Radius (nm)	Rn = 30.5 Rw = 32.1 Rz =35	Rn = 29.7 Rw = 31.4 Rz = 34.9	Rn = 29.5 Rw = 30.9 Rz = 33.5	Rn = 29.1 Rw = 30.6 Rz = 33.7	Rn = 47 Rw = 47.3 Rz = 47.7	Rn = 47.4 Rw = 47.7 Rz = 48.1
OAg/protein ratio		0.8	1.1	2.5	2.7	0.24	0.25
OAg O-acetylation (%)		90.7	88.2	4.1	4.0	-	-

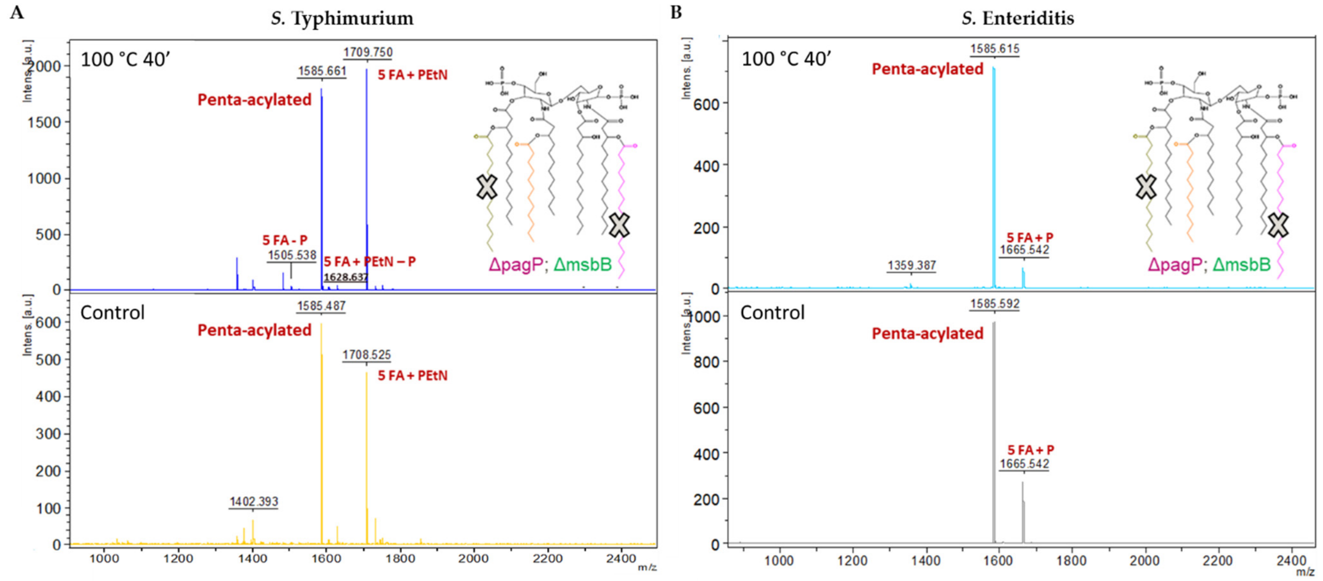

Figure S1. MALDI-TOF-MS analysis on lipid A extracted from *S.* Typhimurium (**A**) and *S.* Enteriditis GMMA (B) after harsh stress at 100 °C for 40 min in comparison to that of control samples: no impact on lipid A structure as the same forms were present in GMMA samples after stress (FA = fatty acids; P = phosphate group; PetN = Phosphoethanolamine). A pyrophosphate linkage is formed where an adduct + P or + PEtN is found.

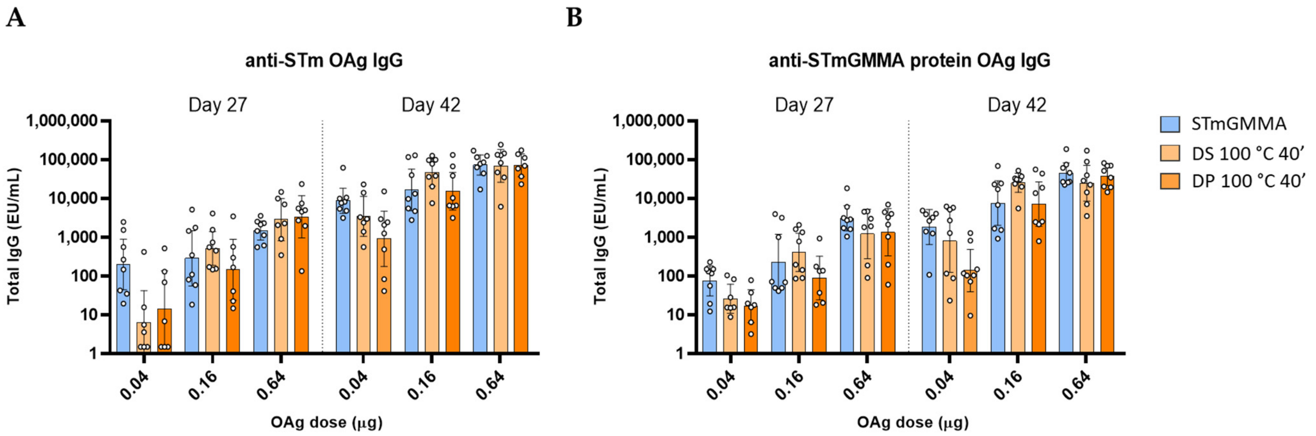

Figure S2. Immunogenicity results from a dose-ranging study in mice with *S.* Typhimurium stressed in harsh temperature conditions: (A) anti-OAg and (B) anti-GMMA protein IgG at Day 27 and Day 42. Graphs report single mice EU/mL (dots), with geometric mean (bars) and 95% confidence interval.

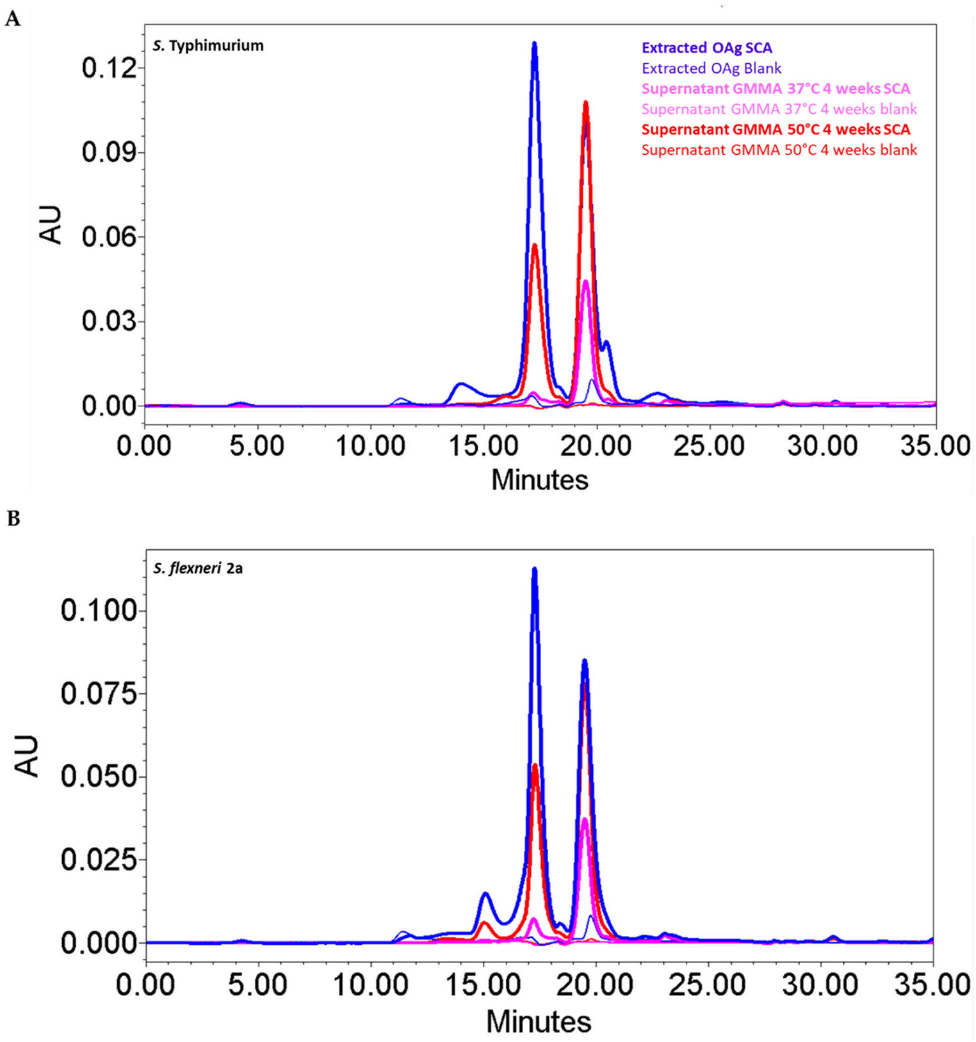

Figure S3. HPLC-SEC analysis with semicarbazide method performed on undiluted supernatants of stressed *S.* Typhimurium (A) and *S. flexneri* 2a GMMA (B): these samples, after derivatization with semicarbazide (SCA), showed an absorption profile at 252 nm matching the ones of the corresponding extracted OAgs (chromatograms in blue), suggesting that OAg found in the supernatants of ultracentrifuged stressed GMMA is released as consequence of the cleavage of the linkage between the KDO and lipid A, and the release increased at 50 °C.

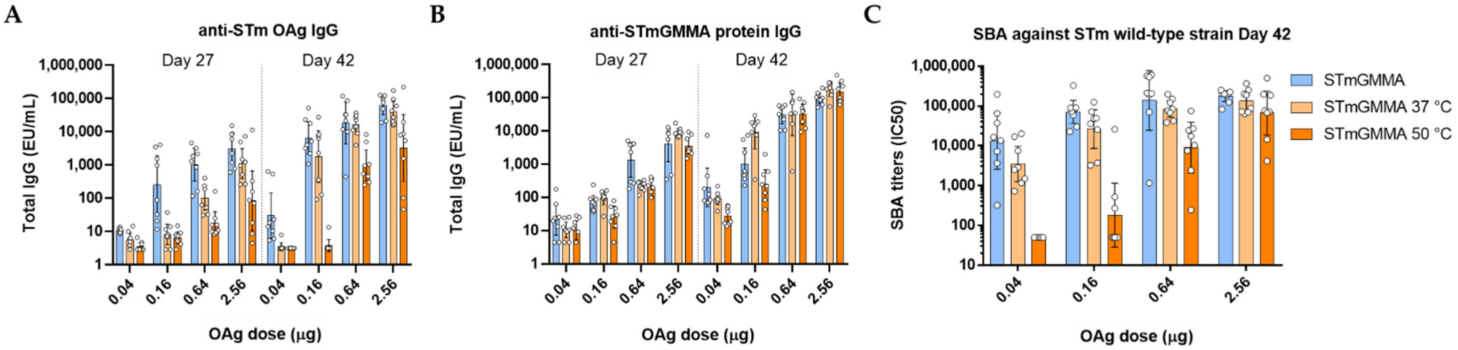

Figure S4. Immunogenicity results from a dose-ranging study in mice with *S.* Typhimurium stressed at 37 °C or 50 °C for 4 weeks in comparison to control GMMA: (**A**) anti-OAg ELISA, (**B**) anti-GMMA protein ELISA and (**C**) SBA against *S.* Typhimurium wild-type strain. Graphs report single mice EU/mL in ELISA or SBA titers (dots), with geometric mean (bars) and 95% confidence interval.

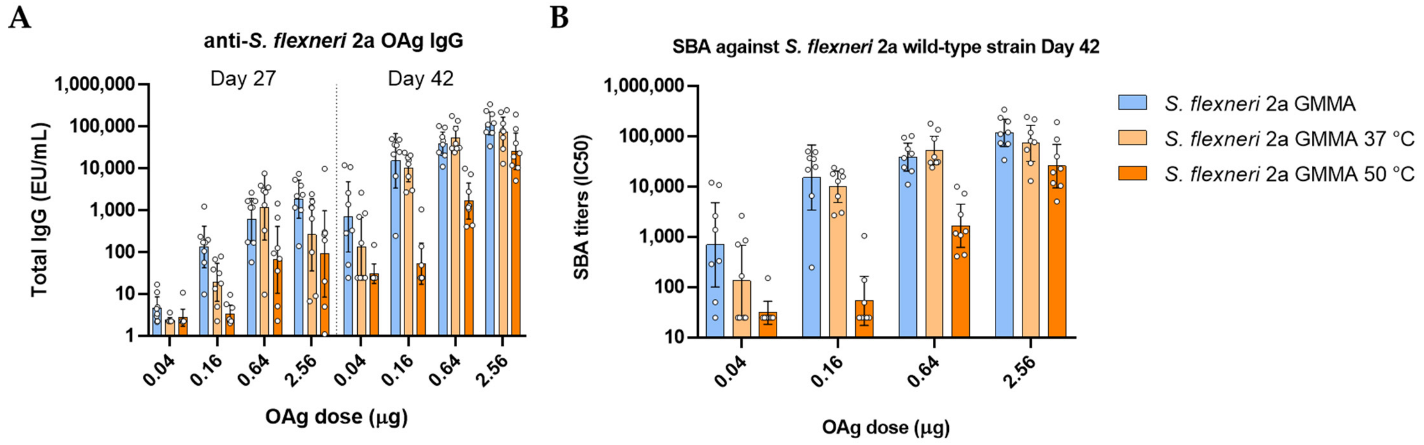

Figure S5. Immunogenicity results from a dose-ranging study in mice with *S. flexneri 2a* GMMA stressed at 37 °C or 50 °C for 4 weeks in comparison to control GMMA: (A) anti-OAg ELISA and (B) SBA against *S. flexneri* 2a wild-type strain. Graphs report single mice EU/mL in ELISA or SBA titers (dots), with geometric mean (bars) and 95% confidence interval.

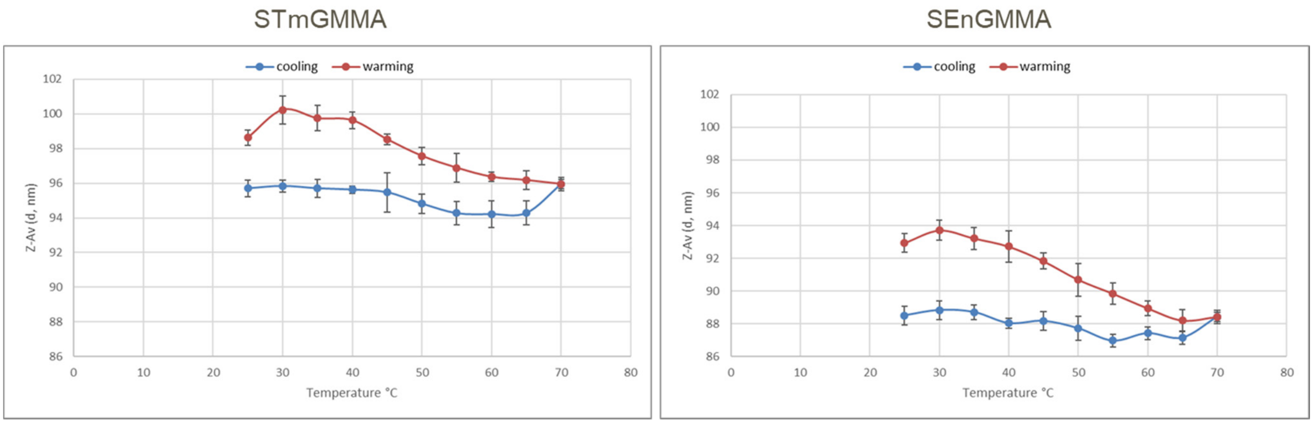

Figure S6. DLS experiment performed warming up *S.* Typhimurium and *S.* Enteriditis GMMA to 70 °C and cooling down to the starting temperature of 25 °C: Z-average decreased from 99 nm to 96 nm in *S.* Typhimurium GMMA and from 93 to 88 in *S.* Enteriditis GMMA. Size distribution changes were not reversible for both types of GMMA. No differences were observed in terms of PDI at all the temperatures.

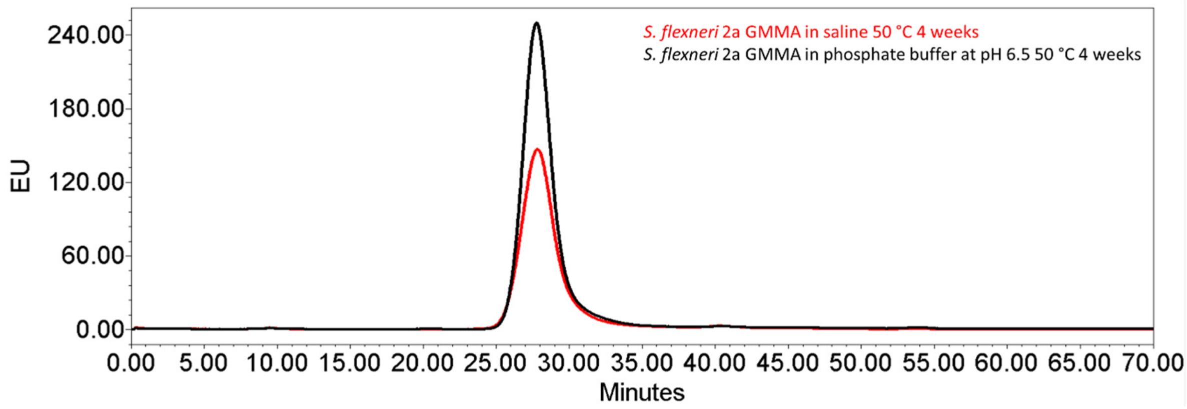

Figure S7. Comparison between HPLC-SEC fluorescence emission profiles of *S. flexneri* 2a GMMA stored in saline or phosphate buffer at pH 6.5, after a 4-weeks incubation at 50 °C: at pH 6.5, fluorescence intensity of GMMA was also impacted but in a lesser extent respect to those stored in saline.

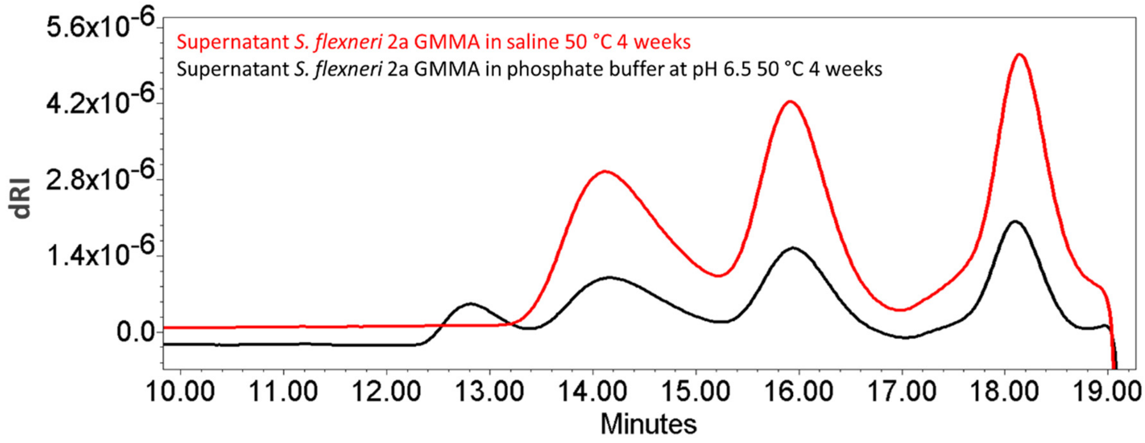

Figure S8. HPLC-SEC analysis of the supernatants of *S. flexneri* 2a GMMA stored in saline or phosphate buffer at pH 6.5, after a 4-weeks incubation at 50 °C: a lower percentage of OAg resulted detached from GMMA membrane at pH 6.5 (samples were analyzed at the same concentration).

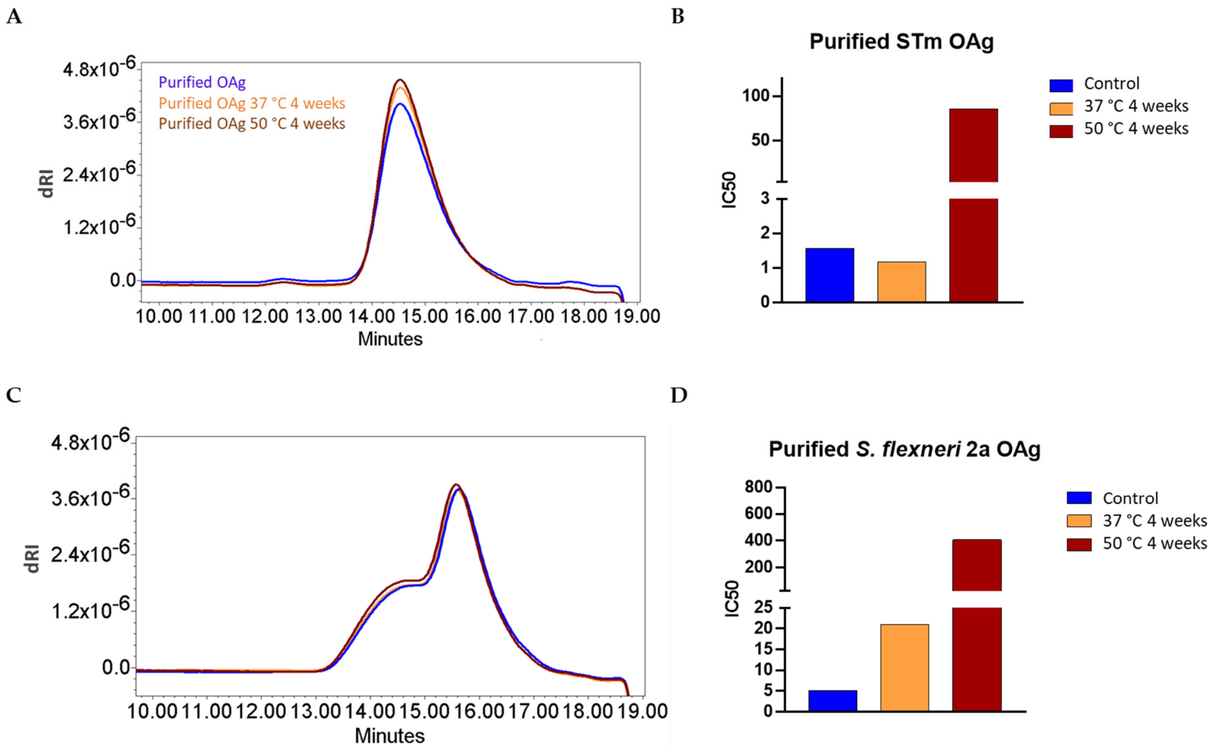

Figure S9. Stability of purified *S.* Typhimurium and *S. flexneri* 2a OAg incubated at 37 °C and 50 °C for 4 weeks: OAg size did not change (A and C, respectively), while loss of mAb recognition was found in cELISA (B and D), reflecting the decrease in the OAg O-acetylation level.

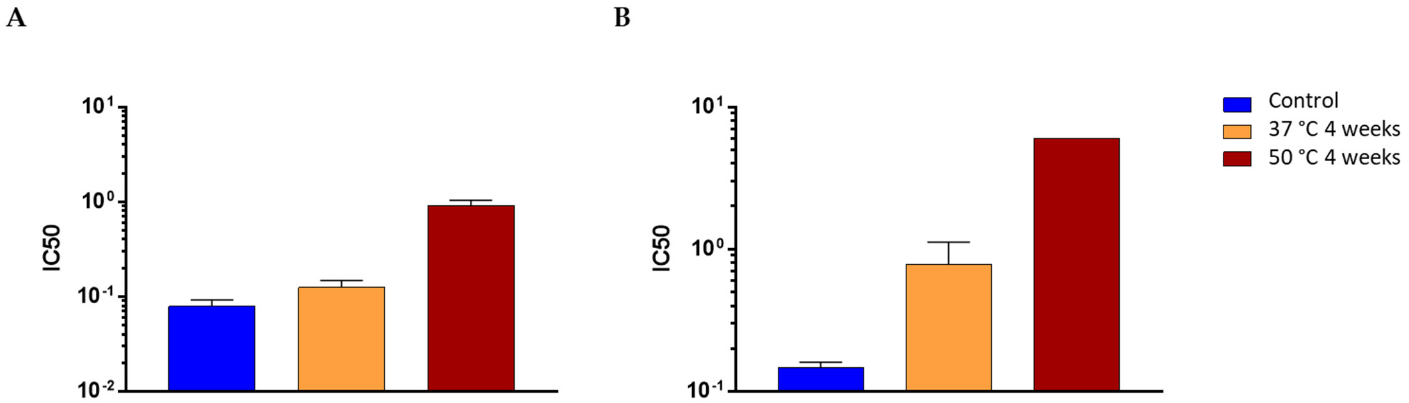

Figure S10. FAcE analyses on *S.* Typhimurium (**A**) and *S. flexneri* 2a GMMA (**B**) formulations stressed at 37 °C and 50 °C for 4 weeks: IC50 increased with temperature, indicating a loss in the recognition by their specific mAb.
